# Enhancing physical fitness using recreational soccer and basketball: A parallel-controlled 8-week study involving overweight and obese individuals, with consideration of sex-related interactions

**DOI:** 10.5114/biolsport.2025.139081

**Published:** 2024-05-24

**Authors:** Qi Xu, Rui Miguel Silva, Piotr Zmijewski, Ting Yu Li, Jian Yong Li, LiuXi Yang, Filipe Manuel Clemente

**Affiliations:** 1 Gdansk University of Physical Education and Sport, 80-336 Gdańsk, Poland; 2 Sport Physical Activity and Health Research & Innovation Center (SPRINT), Rio Maior, Portugal; 3 Escola Superior Desporto e Lazer, Instituto Politécnico de Viana do Castelo, Rua Escola Industrial e Comercial de Nun’Álvares, 4900-347 Viana do Castelo, Portugal; 4 Jozef Pilsudski University of Physical Education in Warsaw, Warsaw, Poland; 5 School of Athletic Performance, Shanghai University of Sport, 200438, Shanghai, China

**Keywords:** Football, Team sports, Physical exercise, Physical fitness

## Abstract

The purpose of this study was to examine the effects of 8-week intervention of recreational soccer (SCG) and basketball (BCG) conditioned games, as compared to self-exercise (SECG) and inactive (ICG) control groups, on aerobic capacity, vertical (VJ) and horizontal jump (SLJ) performance, and handgrip maximal strength (HG) in sedentary overweight and obese men and women. Ninety male and female sedentary overweight and obese volunteers (19.8 ± 1.5 years; 27.9 ± 1.8 m/kg^2^) participated in this experimental parallel controlled design study and were examined on three occasions. Within-group analysis revealed that SCG and BCG significantly (p < 0.05) improved the performances after 8 weeks in the multistage fitness test (MFT) (23.3 and 19.6%, respectively), HG in right (1.6% and 2.9%) and left hands (1.3 and 1.7%), SLJ (5.8 and 1.4%) and VJT (27.4% and 33.9%). Between-group analysis revealed significantly greater post-intervention improvements in SCG (p < 0.001) and BCG (p = 0.043) than ICG in MFT. Improvements in SLJ were greater in SCG (p < 0.001) and BCG (p < 0.001) than ICG, being also better in SCG in comparison to SECG (p = 0.033). VJ performance was significantly better in SCG (p < 0.001), BCG (p < 0.001) and SECG (p = 0.002) than ICG. Only the improvements in HG right (p = 0.042), and SLJ (p = 0.016) showed interactions with sex. This study showed that both SCG and BCG are effective interventions for enhancing health-related physical fitness, specifically in terms of aerobic capacity and strength. Similar benefits in aerobic capacity could be attained through an 8-week self-regulated activity programme, engaging in activity 3 days per week following a supportive lecture on lifestyle change. Considering sex differences, men demonstrated greater improvements in strength and jumping variables when participating in recreational soccer and basketball. On the other hand, women exhibited more significant enhancements in self-selected activities compared to men, particularly in terms of aerobic capacity.

## INTRODUCTION

The growing prevalence of sedentarism, coupled with the higher rates of overweight and obesity, has emerged as a public health concern [1]. Sedentarism is characterized by prolonged periods of sitting and insufficient physical activity, which contribute to a great variety of health-related issues [2]. The sedentarism epidemic also has implications at the socio-economic scale [3]. The sedentary lifestyle extends beyond personal health, including a broader societal dimension that impacts healthcare costs, productivity, and overall health quality [3]. As individuals show sedentary behaviours, the incidence of non-communicable diseases rises exponentially [4]. Cardiovascular diseases, diabetes, and musculoskeletal disorders, among others, are increasingly associated with sedentarism and overweight [4].

The consequences of sedentarism and overweight emphasize the need to educate inactive individuals to follow an active lifestyle [1]. Different physical fitness measures, such as aerobic capacity and muscular strength and power, have a great impact on health adaptations [5]. In fact, sustaining or improving fitness levels is associated with a decreased risk of all-cause and cardiovascular disease (CVD) mortality [6]. Preventing age-related declines in fitness is crucial for promoting longevity, regardless of changes in BMI [7]. Thus, performance in the 20 m Multistage Shuttle Run Test can serve as a valuable indicator of cardiorespiratory fitness and overall health [8, 9]. Moreover, a lower level of muscular strength and power, as measured by jumping performance, may be significantly correlated with subsequent health outcomes [10]. Moreover, low hand-grip strength is also linked to increased hospitalization, nutritional status, overall mortality, and quality of life [11].

While reducing fat mass and increasing muscle mass are crucial for improving muscular performance and overall fitness [12, 13], interesting findings suggest that achieving a high level of fitness, particularly in terms of cardiorespiratory health, may outweigh the importance of fat loss in reducing the risk of cardiovascular disease and all-cause mortality [14]. Therefore, enhancing physical fitness, even without significant changes in body composition, can be an optimal approach to disease prevention and reducing overall mortality [15].

One of the challenges in adhering to a physical exercise routine is maintaining motivation and engagement [16]. Frequently, individual training methods such as running or strength training may not provide the required engagement factors for certain individuals [17]. Thus, engaging in group activities can be one of the recommended ways to promote adherence in overweight and obese subjects [18].

Recreational team sports are gaining popularity as an alternative to individual training methods such as running [19, 20]. Recreational team sports have great benefits, including social interaction, greater motivation to participate, and competition [19]. Team sports, in general, are characterized by intermittent bouts of high-intensity efforts interspersed with periods of lower intensity [21, 22]. Engaging in recreational team sports shows significant improvements in body composition [23], maximum aerobic power, blood pressure, muscle capillarization, and performance improvement [24]. These effects align with those observed in running- and strength-based interval training [25, 26].

Recreational small-sided games, particularly as training drills, offer significant benefits. They foster more homogeneous participation among individuals, thereby enhancing individual technical skills and involvement in various playing scenarios [27]. Additionally, they ensure high intensity levels, typically ranging from 82 to 84% of maximal heart rate, across formats ranging from 3 v 3 to 7 v 7 [28]. Moreover, these games have a notable impact on locomotor demands, such as total distance covered, high-intensity movements, and the frequency of accelerations and decelerations [28]. Ultimately, this can lead to greater muscular engagement [29] and improvements in overall physical fitness.

Despite this, a gap exists in comparative studies exploring differences among recreational team sports [26, 30, 31]. Frequently, the studies report only one team sport (most often soccer) for comparison against alternative individual training methods or passive controls [32, 33]. This approach limits the opportunity to gain a comprehensive understanding of whether different team sports (e.g., basketball, handball) can induce distinct physical adaptations [34]. Team sports come with their own physiological and physical demands, and exploring various options such as small-sided games could provide valuable insights.

Moreover, the predominant focus within the existent body of literature is on differentiated demographic cohorts, such as older adults or children [11]. The scarcity of studies investigating the impact of recreational team sports on young adults is typically more pronounced, thereby constraining our understanding of the associated effects and benefits. Furthermore, experimental studies frequently combine results without distinguishing between sexes [35, 36] or report a single sex [37], making it uncommon to observe how recreational team sports might induce distinct physical fitness adaptations based on sex.

Recognizing the significance of comparing diverse recreational team sports in relation to physical fitness adaptations among over-weight and obese individuals, and acknowledging sex as a potential interacting factor influencing the magnitude of these adaptations, this study aimed to investigate the impact of recreational soccer and basketball-conditioned games. The comparison included a self-exercise group and an inactive group, with a focus on aerobic capacity, vertical and horizontal jump performance, and handgrip maximal strength in both sedentary men and women.

## MATERIALS AND METHODS

### Study design

This study is a parallel controlled study design, in which two experimental groups (exposed to soccer conditioned games [SCG] and basketball conditioned games [BCG]) were compared to two control groups (one self-exercise group [SECG] in which participants engaged in exercise by self-prescribing themselves and one inactive control group [ICG] in which subjects did not engage in physical exercise). The study had three moments of assessment (pre-intervention, week 0; mid-intervention, week 4; post-intervention, week 9). The intervention period lasted 8 consecutive weeks, in which participants from SCG and BCG were exposed to three weekly sessions (n = 24).

Convenience sampling was used. The recruitment process started by inviting young adults from Chendu city who were overweight (body mass index, BMI:25.0–29.9 kg/m^2^) or obese (BMI ^3^30 kg/m^2^) by means of social media groups and public announcements. After receiving the interest of participants, they were matched with the eligibility criteria. Eligibility criteria was a priori established based on the following items: (i) having a BMI > 25 kg/m^2^; (ii) being sedentary and not engaging in regular physical exercise; and (iii) not presenting an injury or chronic diseases at the time of the experiments. An additional criterion was used for further data treatment: the participant was required to have an adherence rate of over 80% in the case of experimental SCG and BCG groups and participate in all three assessment time points.

After the selection of the participants, they were randomly assigned to one of the SCG, BCG or control groups by simple randomization. The randomization was made by sealed opaque envelopes, and before the first assessment was made. Allocation was concealed, in which the person who checked the volunteers against inclusion criteria was not aware of the subsequent allocation to the groups. After the first assessment, it was observed that the groups were not significantly different for the main physical fitness variables (p > 0.05).

The study was not blind to the participants and the coaches who administered the training sessions. However, assessors during the three times of evaluation were blind to the groups and participants.

### Ethical aspects

The study design was preliminary approved by the Chendu Institute of Physical Education with the code number 124/2023. The study respected the ethical standards for the study in humans as defined by the Declaration of Helsinki.

### Participants

Using an effect size of 0.13 [38], for 4 groups, a power of 0.85 and 1 covariate, G*Power (version 3.1.9.6., Heinrich-Heine-Universität Düsseldorf, Düsseldorf, Germany) identified a recommended number of 87 participants. Among the 98 volunteers who expressed their availability to participate in the study, 90 were selected while the remaining 8 were excluded because they had chronic diseases (e.g., hypertension, diabetes). Overall, participants were 19.8 ± 1.5 years old, 45 men and 45 women, BMI of 27.9 ± 1.8 m/kg^2^, body mass 75.1 ± 8.5 kg and height of 1.68 ± 0.07 m.

After being randomly allocated to groups, the characteristics of each group were as follows: SCG, n = 30, 15 men and 15 women, BMI 27.7 ± 1.4 m/kg^2^; BCG, n = 30, 15 men and 15 women, BMI 28.0 ± 2.0 m/kg^2^; control group, n = 30; 15 men and 15 women, BMI 27.3 ± 4.2 m/kg^2^. Of note, within the control group, ten participants were classified as SECG (comprising 5 men and 5 women; 27.1 ± 1.7 m/kg^2^), whereas 20 participants were categorized as ICG (including 10 men and 10 women; 28.3 ± 1.8 m/kg^2^).

Among the 90 participants, 11 individuals (12.2%) were identified as obese, with a BMI equal to or greater than 30 kg/m^2^. Specifically, there were 2 obese individuals in the SCG, both of whom were men, 4 in the BCG (3 men and 1 woman), 1 man in the SECG, and 4 in the ICG (1 man and 3 women). Following the intervention, the groups exhibited the following characteristics: SCG, n = 30, 15 men and 15 women, mean BMI 25.1 ± 1.6 kg/m^2^; BCG, n = 30, 15 men and 15 women, mean BMI 25.4 ± 2.1 kg/m^2^; and the control group, n = 30, 15 men and 15 women, mean BMI 26.1 ± 2.1 kg/m^2^.

The participants were assessed for their daily physical activity patterns using the short-version International Physical Activity Questionnaire at three time points (pre-, mid- and post-intervention). Considering the reports, the participants of control groups who ful-filled the World Health Organization recommendations for being physically active over the experimental period were classified as the self-exercise control group (SECG) while the others were classified as the inactive control group (ICG). The adherence rate of participants in the SCG was 95% and in the BCG 97%. After allocation to groups, no dropout was registered. Attendance was recorded for all sessions by marking participant presence. However, no specific encouragement or engagement strategies were implemented beyond the participants’ inherent commitment to the training.

### Training intervention

The SCG and BCG training sessions were conducted three times per week, with a standardized warm-up duration of 10 minutes preceding each session. The warm-up comprised five minutes of self-regulated jogging followed by five minutes of targeted lower-limb dynamic stretching, with a focus on the hamstrings, adductors, abductors, quadriceps, and calves. For SCG, two different formats were employed: a 2 v 2 format and a 4 v 4 format. In the 2 v 2 format, four sets of 3 minutes were played, with a 2-minute rest interval between sets. The games were conducted using a small goal of 2 m, and there was no goalkeeper. The absence of an offside rule contributed to a dynamic and fluid gameplay. The ball replacement was executed with the foot, ensuring a continuous and fast-paced game. The field dimensions for the 2 v 2 format were 25 × 15 m. In the 4 v 4 format, four sets of 5 minutes were played, with a 2-minute rest period between sets. Similar to the 2 v 2 format, the game was played with a small goal of 2 m, no goalkeeper, and no offside rule. The field dimensions for the 4 v 4 format were slightly larger, measuring 35 × 20 m.

In the case of BCG, the training sessions also took place three times per week, and a 5-minute warm-up preceded each session. Two different formats were utilized: a 3 v 3 format and a 5 v 5 format. For the 3 v 3 format, four sets of 3 minutes were played, with a 2-minute rest period between sets. The games were played using the regular target, and the field dimensions for the 2 v 2 format were set at a quarter court size. In the 5 v 5 format, four sets of 5 minutes were played, with a 2-minute rest interval between sets. Similar to the 3 v 3 format, the games were played using the regular target. The field dimensions for the 3 v 3 format were set at half of the court. These standardized training sessions and game formats were employed to ensure consistency and facilitate the comparison of out-comes between the two recreational conditioned.

The rationale behind employing small formats (smaller than 5 v 5) stems from scientific evidence indicating that these formats often elevate overall physiological intensity, leading to increased time spent in higher heart rate intensity zones, thereby favouring aerobic power development [39]. Additionally, these smaller formats are associated with greater individual movement (total distance covered), which can ultimately contribute to muscular improvements [40] and enhance technical engagement [27]. Furthermore, the formats utilized in our study align with the methodological approaches of previous experimental studies on recreational small-sided games conducted in sedentary populations [41].

The intervention sessions were conducted separately for men and women, scheduled at different times. This approach facilitated the management of proficiency levels and ensured a varied stimulus impact. Given the diverse formats of the SSGs, participants were organized into teams and rotated accordingly, allowing for adequate recovery and work periods. This strategy also ensured the involvement of the necessary number of players in the games. Management was carried out with the aim of providing balanced playing time for all participants.

Participants’ exertion levels during training sessions were assessed using the Borg CR 10 Scale, with individual scores recorded approximately 30 minutes after the conclusion of each session. The scale was utilized because it encompasses both the cardiorespiratory and muscular efforts associated with exercise [42]. Furthermore, heart rate was monitored at a frequency of 1 Hz using a telemetry-based heart rate monitor (Polar RS400, Kempele, Finland).

Prior to the commencement of the experimental study, all participants were introduced to a lecture centred on promoting an active lifestyle, general dietary regulation, and understanding their longterm health impacts. This lecture offered only general tips regarding the importance of exercise and adopting a healthy, active lifestyle, with no specific training or dietary plans provided. After attending this lecture, a group of 10 participants (5 men and 5 women) from the control group spontaneously and independently began to adopt an active lifestyle and engage in physical exercise. This commitment was observed during the periodic assessments of physical activity patterns conducted midway and at the conclusion of the intervention. Throughout the experimental period of the intervention, neither the experimental nor the control groups received any dietary plans, or physical activity individualized guidance.

Individuals in the control group who opted for self-exercise did so voluntarily, spontaneously, and independently, without any intervention from the research team or other parties, such as personal trainers. All the participants underwent assessments three times during the study period to monitor their reported weekly physical activity, utilizing the short-version International Physical Activity Questionnaire. The responses enabled the classification of participants into active and inactive categories. Those active in the SECG had the freedom to make self-prescriptions, selecting their preferred activities, including running, swimming or resistance training, among others. The requirement stipulated that participants in the control group must engage in self-prescribed activities, prohibiting the utilization of personal training services or physical classes. This stipulation aimed to ensure that individuals remained solely responsible for their voluntary exercise. These requirements were included in the criteria for participating as a volunteer in the study. Participants were informed that they could withdraw at any time without facing penalties. Additionally, if needed, participants could seek guidance from the research team regarding their lifestyle and spontaneous exercise, although this did not include any training or dietary plans.

### Methodological procedures

At the three evaluation points (pre-, mid-, and post-intervention), participants were assessed on the same day of the week (Mondays), and in all cases, they were instructed to have a rest period of 48 hours before the evaluation. The physical fitness assessments occurred by the morning, ~9 a.m., in an indoor facility with a temperature of 19.6 ± 1.2ºC and relative humidity of 47.3 ± 4.3%. The assessments started with the response to demographic questionnaire and short-version International Physical Activity Questionnaire. Afterwards, the participants underwent anthropometric measurements before proceeding to a standardized warm-up protocol. This protocol comprised 5 minutes of self-regulated jogging, followed by 5 minutes of active mobilization of the lower limbs, and concluded with 5 minutes of dynamic stretching for the lower limbs. Following the completion of the warm-up, they underwent a sequence of assessments, beginning with the maximal strength handgrip test, followed by the vertical jump and standing long jump, and concluding with the 20 m multistage fitness test. Between the handgrip and vertical jump tests, participants rested for 3 minutes, while a rest period of 5 minutes was implemented between the subsequent tests to ensure complete recovery, with a specific focus on the tests involving lower limbs. The order of the tests and the duration of the rest periods remained consistent for all participants.

### Anthropometric assessment

Ensuring proper alignment, participants were directed to stand with their backs against a stadiometer and look straight ahead to achieve a horizontal alignment of the Frankfort plane. Positioned in front of the participants, the assessors then proceeded to adjust the stadiometer marker (ADE MZ10042, ADE, Germany) for precise measurements. For body mass assessment, participants stood facing forward on the platform’s centre. Utilizing an electronic flat scale (Model 813, SECA, Germany), measurements were conducted.

### Handgrip strength

The assessment of handgrip strength utilized the TKK dynamometer (TKK 5101 Grip-D, Takey, Tokyo, Japan). A consistent error of 0.49 kg was associated with the instrument [43]. Participants were instructed to extend their arms, employing their dominant hand to exert maximum force on the dynamometer. This grip had to be sustained for a minimum of two seconds, and the procedure was repeated three times, with the peak force documented for subsequent analysis.

Each participant underwent three trials per hand, interspersed with a 2-minute rest between trials. The trial yielding the highest recorded maximal strength was selected for statistical treatment. The mean coefficient of variation for within-participant analysis, indicating variability between trials, stood at 1.9% in handgrip strength.

The primary outcome extracted was the maximal handgrip strength in both left and right hands, with the highest values being extracted for each limb.

### Vertical jump and standing long jump

For the vertical jump assessment, participants were guided to begin from a squat-jump position with a 90° knee flexion, maintaining a shoulder-width stance with hands on their hips [44]. The instruction was to execute a jump aiming for maximum height, all while keeping their hands on their hips throughout. In the case of the standing long jump, participants were positioned at the start line and instructed to leap horizontally, attempting to cover the greatest distance while adhering to the guideline of keeping hands on hips. Swinging arms during the jump was expressly prohibited, and successful jumps necessitated a landing on both feet without subsequent corrective movements [45]. These jumps have previously been utilized in overweight populations as a means to assess muscular power [46].

The My Jump 2 app on iPhone X was used to quantify the height and length of jumps. The measurement of jump height and length was conducted using the My Jump 2 app on an iPhone X. The mobile app calculates jump height by capturing both the take-off and landing phases, utilizing the equation *h* = *t*^2^ × 1.22625, where *h* represents the jump height in metres, and *t* denotes the flight time in seconds [47]. This choice was based on the demonstrated validity and reliability of My Jump 2 in assessing both jump height and jump length [47]. Each participant executed three trials for both the vertical and standing long jumps, with a 2-minute interval between each trial. The within-participant analysis, reflecting variability between trials, revealed an average coefficient of variation of 2.9% for the vertical jump and 3.2% for the standing long jump. The highest recorded vertical jump (in centimetres) and the longest standing long jump (in metres) were selected for subsequent data analysis.

### 20 m multistage fitness test

Participants underwent the 20 m shuttle run test in accordance with the established protocol outlined in a prior publication [48]. The test has previously been reported for its reproducibility among obese populations [49]. The test involved participants running back and forth between two lines spaced 20 m apart, employing a pivot around a central point positioned between these lines. Participants were granted the option to pivot and continue running within the designated boundaries instead of physically crossing the line before turning. The participants’ pace was regulated by an audio beep harmonized with the standard velocity progression associated with the test [48].

The test was concluded under two circumstances: either the participant failed to reach a line following two consecutive beeps, or the participant omitted waiting for the beep twice consecutively. The primary outcome was determined by recording the total distance covered by each participant during the shuttle run test.

### Statistical procedures

Descriptive statistics were presented in the form of the mean, standard deviation, and percentage difference, calculated as post-pre/pre´100. Normality and homogeneity of the data were explored and confirmed using the Kolmogorov-Smirnov test (p > 0.05) and Levene’s test (p > 0.05), respectively. Descriptive statistics are presented in the form of mean and standard deviation. Analysis of covariance (ANCOVA) for repeated measures adjusted for sex was used for within- and between-group comparisons. Partial eta squared was used for reporting ANCOVA effect size, and Cohen’s d for reporting pairwise comparisons. The Bonferroni post hoc test was also used as a post hoc test. Furthermore, a contrast analysis (utilizing the mean post-pre difference) was conducted as part of a two-way ANOVA examining the interaction between group and sex. Statistical tests were executed using the software SPSS (version 29.0.0., IBM SPSS Statistics, Armonk, NY: IBM Corp) with a significance level of p < 0.05.

## RESULTS

### Baseline assessments

Non-significant differences at baseline were found between groups considering total distance covered in the 20 m multistage fitness test (p = 0.773; = 0.013), handgrip strength right hand (p = 0.145; = 0.063), handgrip strength left hand (p = 0.125; = 0.067), standing long jump (p = 0.487; = 0.029) and vertical jump (p = 0.212; = 0.053).

### Training load monitoring

**FIG. 1:**
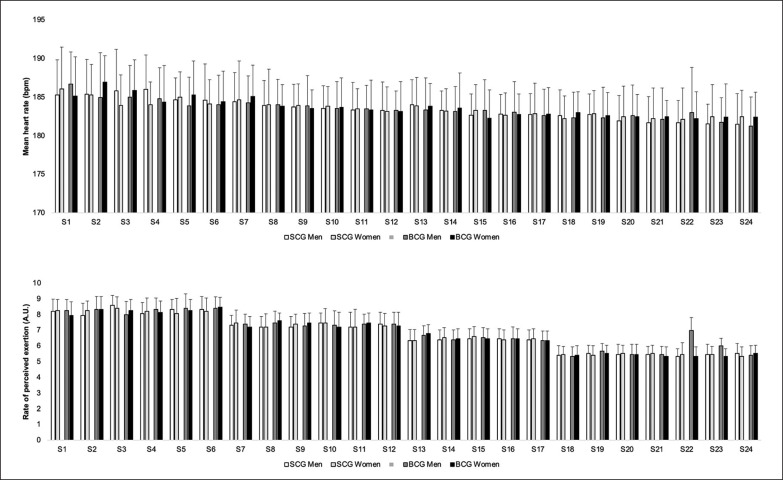
Descriptive statistics (mean and standard deviation) of mean heart rate and perceived exertion levels among volunteers in the SCG and BCG groups throughout the experimental sessions.

Men participants in the SCG and BCG showed mean heart rate values of 183.5 ± 1.4 bpm and 183.4 ± 1.2 bpm, respectively, while women participants had values of 183.5 ± 1.0 bpm and 183.6 ± 1.3 bpm, respectively. Figure 1 shows the descriptive statistics of mean heart rate and perceived exertion levels among volunteers in the SCG and BCG groups throughout the experimental sessions. Interestingly, it was observed that the mean heart rate for the first 6 sessions among all participants in both the SCG and BCG was 185.1 ± 0.9 bpm, decreasing to 183.8 ± 0.5 bpm in the second cluster of 6 sessions, further reducing to 183.0 ± 0.5 bpm in the third cluster, and finally reaching 182.2 ± 0.5 bpm in the last cluster of 6 sessions.

The SECG demonstrated an average of 2.2 days engaging in moderate activities, with an average duration of 51.2 ± 8.9 minutes per day. Additionally, there was an average of 0.9 ± 0.3 days dedicated to vigorous physical activities, during which participants spent an average of 34.3 ± 5.6 minutes.

### Pre-to-post training variations

Table 1 shows the descriptive statistics of the two experimental and the two control groups over the periods of the experiment (pre-, mid- and post-intervention). Within-group analysis revealed that SCG significantly improved the performances after 8 weeks in the multistage fitness test (23.3%; p < 0.001; d = 0.669), handgrip maximal strength at right (1.6%; p < 0.001; d = 0.072) and left hands (1.3%; p < 0.001; d = 0.063), standing long jump (5.8%; p < 0.001; d = 0.710) and vertical jump (27.4%; p < 0.001; d = 0.807).

**TABLE 1:** Descriptive statistics (mean ± standard deviation) and inferential statistics for the within- and between-group comparisons for the overall participants.

	SCG (n = 30)	BCG (n = 30)	SECG (n = 10)	ICG (n = 20)	Mixed repeated measures ANCOVA
**20 m MFT (m)**					
Pre	426.0 ± 148.9^$,#^	404.0 ± 150.3^$,#^	412.0 ± 136.0^$,#^	415.0 ± 131.5	time*group*sex (F = 1.704; p = 0.147; = 0.059)
Mid	472.0 ± 139.5^*,#^	440.0 ± 155.3^*,#^	468.0 ± 137.0^*,#^	432.0 ± 131.1	time*sex (F = 1.774; p = 0.182; = 0.021)
Post	525.3 ± 148.2^*,$^	483.3 ± 158.4^*,$^	504.0 ± 111.9^*,$^	416.0 ± 126.3	time*group (F = 2.603; p = 0.034; = 0.087)
Post-pre (%D)	Ý 23.3%	Ý 19.6%	Ý 22.3%	Ý 0.2%	

**HG right (kg)**					
Pre	31.2±6.7^$,#^	30.9±5.6^$,#^	28.3±6.4^$,#^	30.0±4.6^$,#^	time*group*sex (F = 3.321; p = 0.018; = 0.108)
Mid	31.4 ± 6.8^*,#^	31.4 ± 5.7^*,#^	28.6 ± 6.4^*,#^	30.1 ± 4.6^*,#^	time*sex (F = 4.025; p = 0.042; = 0.047)
Post	31.7 ± 6.9^*,$^	31.8 ± 5.8^*,$^	28.9 ± 6.4^*,$^	30.2 ± 4.6^*,$^	time*group (F = 7.063; p < 0.001; = 0.205)
Post-pre (%D)	Ý 1.6%	Ý 2.9%	Ý 2.1%	Ý 0.7%	

**HG left (kg)**					
Pre	30.4±6.8^$,#^	29.9±5.7^$,#^	27.3 ± 6.5^#^	29.0 ± 4.5	time*group*sex (F = 2.085; p = 0.096; = 0.071)
Mid	30.5 ± 6.9^*,#^	30.1 ± 5.6^*,#^	27.4 ± 6.4	29.0 ± 4.5	time*sex (F = 0.384; p = 0.574; = 0.005)
Post	30.8 ± 6.9^*,$^	30.4 ± 5.7^*,$^	27.7 ± 6.4^*^	29.0 ± 6.0	time*group (F = 3.845; p = 0.008; = 0.123)
Post-pre (%D)	Ý 1.3%	Ý 1.7%	Ý 1.4%	Þ 0.0%	

**SLJ (m)**					
Pre	1.73 ± 0.11^$,#^	1.72 ± 0.13^$,#^	1.70 ± 0.08^$,#^	1.72 ± 0.11^$^	time*group*sex (F = 2.029; p = 0.094; = 0.069)
Mid	1.79 ± 0.11^*,#^	1.77 ± 0.14^*,#^	1.72 ± 0.08^*,#^	1.73 ± 0.11^*^	time*sex (F = 5.127; p = 0.016; = 0.059)
Post	1.83 ± 0.13^*,^$	1.82 ± 0.15^*,$^	1.76 ± 0.08^*,$^	1.73 ± 0.13	time*group (F = 12.389; p < 0.001; = 0.312)
Post-pre (%D)	Ý 5.8%	Ý 5.8%	Ý 3.5%	Ý 0.6%	

**VJ (cm)**					
Pre	21.9±7.4^$,#^	23.3±5.2^$,#^	24.3±4.5^$,#^	20.7 ± 6.3	time*group*sex (F = 5.157; p < 0.001; = 0.159)
Mid	24.7 ± 7.4^*,#^	27.1 ± 5.5^*,#^	26.5 ± 4.4^*,#^	20.7 ± 6.0	time*sex (F = 2.218; p = 0.126; = 0.026)
Post	27.9 ± 7.6^*,$^	31.2 ± 6.0^*,$^	28.3 ± 4.2^*,$^	20.7 ± 5.9	time*group (F = 22.246; p < 0.001; = 0.449)
Post-pre (%D)	Ý 27.4%	Ý 33.9%	Ý 16.5%	Þ 0.0%	

SCG: soccer conditioned games; BCG: basketball conditioned games; SECG: self-exercise control group; ICG: inactive control group; MFT: multistage fitness test; HG: handgrip strength test; SLJ: standing long jump; VJ: vertical jump;

* : significantly (p < 0.05) different from pre-intervention;

$ : significantly (p < 0.05) different from mid-intervention;

# : significantly (p < 0.05) from post-intervention

Similarly, within-group analysis revealed that BCG significantly enhanced performances after 24 sessions in the multistage fitness test (19.6%; p < 0.001; d = 0.511), handgrip maximal strength in right (2.9%; p < 0.001; d = 0.142) and left hands (1.7%; p < 0.001; d = 0.097), standing long jump (1.4%; p < 0.001; d = 0.582) and vertical jump (33.9%; p < 0.001; d = 1.326).

The SECG significantly improved performances in the multistage fitness test (22.3%; p = 0.044; d = 0.731), handgrip maximal strength in right (+2.1%; p < 0.001; d = 0.094) and left hands (6.2%; p < 0.001; d = 0.055), standing long jump (3.5%; p < 0.001; d = 0.771) and vertical jump (16.5%; p < 0.001; d = 0.862). The within-group analysis revealed that the ICG did not show significant variation in performance in the multistage fitness test (0.2%; p = 0.954; d = 0.008), handgrip maximal strength in left hand (0.0%; p = 0.740; d = 0.007), standing long jump (0.6%; p = 0.199; d = 0.051) and vertical jump (0.0%; p = 0.952; d = 0.002), although significant improvements were found for handgrip maximal strength in the right hand (0.7%; p = 0.010; d = 0.050).

Between-group analysis revealed significantly greater post-intervention performances of SCG (p < 0.001) and BCG (p = 0.043) than ICG in the multistage fitness test, although no significant differences between groups were found for handgrip maximal strength in right (p = 0.088) and left hands (p = 0.071). Standing long jump after intervention was significantly better in SCG and BCG than ICG (p < 0.001 and p < 0.001 respectively), being also better in SCG in comparison to SECG (p = 0.033). Vertical jump performance was significantly better in SCG (p < 0.001), BCG (p < 0.001) and SECG (p = 0.002) than ICG, although no significant differences (p > 0.05) were observed among SCG, BCG, and SECG.

**FIG. 2:**
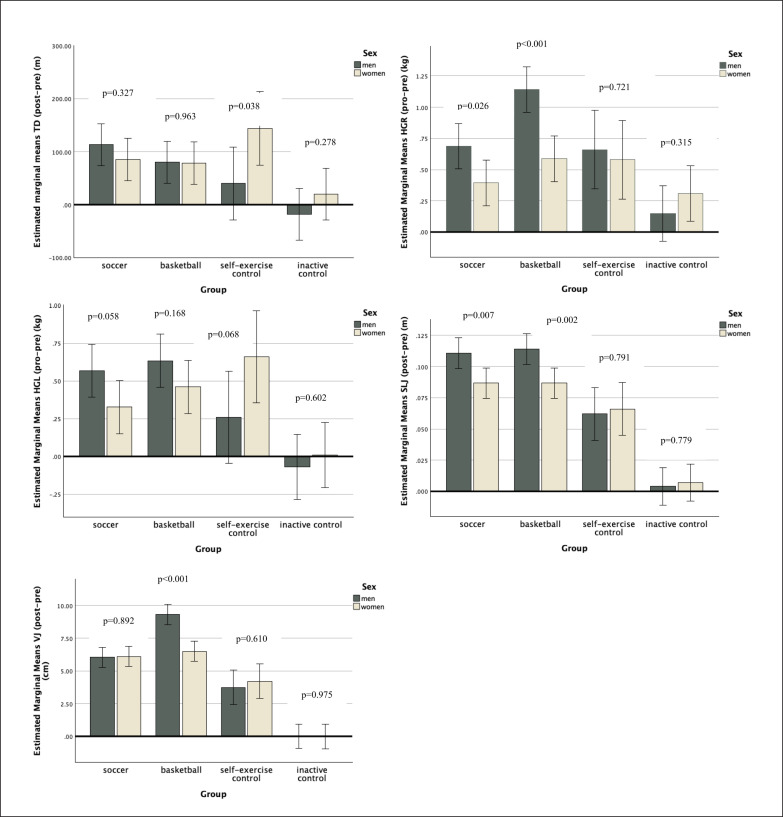
Descriptive statistics (mean ± standard deviation) for contrast analysis (post-pre) physical fitness variables considering groups and sex. * significantly different between sexes at p < 0.05; TD: total distance in multistage fitness test; HG: handgrip strength test for left (L) and right (R); SLJ: standing long jump; VJ: vertical jump VJ: vertical jump.

Figure 2 presents the contrast analysis (post-pre) of physical fitness of men and women considering the experimental and control groups. Significant differences between sexes were found in SECG considering the multistage fitness test (men-women, mean difference of -104 m; p = 0.038; = 0.052). Regarding the right handgrip strength, significant differences between sexes were found in SCG (men-women, mean difference of 0.293 kg; p = 0.026; = 0.059) and BCG (men-women, mean difference of 0.553 kg; p < 0.001; = 0.183). Considering standing long jump, significant differences between sexes were found in SCG (men-women, mean difference of 0.024 m; p = 0.007; = 0.085) and BCG (men vs. women, mean difference of 0.027 m; p = 0.002; = 0.107). As regards vertical jump, significant differences between sexes were found in BCG (men-women, mean difference of 2.809 cm; p < 0.001; = 0.247). No other significant differences were found.

## DISCUSSION

The present study aimed to examine the effects of recreational soccer and basketball conditioned games, self-exercise, and inactive groups, on aerobic capacity, jump performance, and handgrip maximal strength in sedentary men and women overweight and obese individuals. The main findings revealed that, in comparison to the inactive control group, both the soccer and basketball groups exhibited more substantial enhancements in aerobic and jumping performance. While the self-exercise control group demonstrated significant improvements, the structured soccer and basketball groups experienced even greater improvements in vertical jumping. Interaction effects between time and sex were also identified for handgrip strength in the right hand and standing long jump.

In our study, the soccer group improved their aerobic performance by 23.3%, the self-exercise group improved by 22.3%, and the basketball group by 19.6%. This aligns with previous evidence that showed that practising recreational team-based sports improves aerobic capacity [50]. Specifically, a previous systematic review with meta-analysis showed that individuals participating in recreational soccer had large improvements in maximum oxygen uptake (by 3.51 mL/kg/min; ES = 1.46), when compared to strength training and no exercise, regardless of age, sex, and health status [51]. Consequently, overall cardiovascular health is significantly improved in individuals participating in recreational team sports [52]. These improvements may stem from the physiological stimulation triggered by recreational team sports, specifically structured as conditioned games with smaller formats and pitch sizes. This design leads to heightened metabolic demands, as demonstrated by previous studies on small-sided games. Smaller formats ranging between 2 v 2 and 4 v 4 create a greater metabolic power demand, likely due to the increased frequency of accelerations and decelerations and individual actions [39]. Conversely, larger formats of play are more suitable for increasing running demands in high-intensity running, albeit with-out significantly impacting the physiological stimulus regarding aerobic power [39]. The diverse combination of high intensity and varying durations of conditioned games induces adaptations in muscle mitochondria, enhancing their efficiency in utilizing oxygen for energy production [53].

Furthermore, the dynamic and high-intensity characteristics inherent in conditioned games necessitate an augmented supply of oxygen to engaged muscles, resulting in an increased cardiac output. This heightened demand is reflected in an elevated heart rate, signalling the intensified exertion of the heart to fulfil metabolic needs. [54]. Through regular exposure to these stimuli, the cardio-vascular system undergoes adaptations, manifesting as enhancements in both stroke volume and the efficiency of cardiac output and ultimately in improving aerobic capacity [55].

Significant improvements in handgrip strength were found for both right and left hands in the soccer and basketball groups. Contrary to our findings, a previous study conducted on 30 older adults showed that after a 12-week recreational soccer intervention, there were no significant improvements in handgrip strength and no significant differences between the experimental group and the inactive group [56]. However, this evidence discrepancy may be related to the fact that our sample was composed of young adults (~19 years old), and the mentioned study’s sample [56] comprised older adults (> 60 years old). The likelihood that older participants may be experiencing sarcopenia could constrain their ability to increase lean mass and, consequently, enhance their strength performance.

In this line, the increases in handgrip strength observed in the present study, for both soccer and basketball groups, could be related to the strength component of changes of direction, shots, tackles, and jumps during training [57]. Overall strength is critical for daily activities, particularly in overweight and obese individuals. However, comparing these results with other forms of resistance training could provide a greater view of the potential of recreational team sports as an alternative to conventional strength training.

Considering vertical and horizontal performance, our study showed that the SCG, BCG, and SECG groups improved standing long jump and vertical jump performance, while the ICG did not show any improvements. A previous study conducted on 49 inactive individuals revealed that a recreational soccer intervention had no effects on vertical jump performance, which is in contrast with our findings [58]. However, another study reported that an intervention consisting of 64 weeks of recreational conditioned soccer games led to improvements in jump performance, with 4 cm improvement being observed from the 12^th^ to the 64^th^ week of the intervention [59].

The improvements in both vertical and long jump performance noted in soccer and basketball groups can be attributed to the muscular recruitment and movements intrinsic to these sports. Typically, conditioned games involve rapid acceleration, deceleration, and changes of direction, thereby accentuating power muscle recruitment and potentiation [60]. It is acknowledged that strength training interventions show significantly greater magnitudes of change in jump performance when compared to recreational team-based sports alone [58, 61]. However, that does not mean that recreational team sports such as soccer and basketball cannot be used to ensure positive adaptations for jump performance. Indeed, extensive literature indicates that recreational team-based sports significantly increase leg lean mass and jump performance [24, 52, 56, 57]. Moreover, studies have consistently shown a positive correlation between improvements in BMI and improvements in strength [62]. This relationship is primarily attributed to the greater muscle mass [63] typically associated with better BMI levels. Muscle mass is a key determinant of strength, as muscles generate force during contraction [64].

Upon analysing group comparisons, a significant interaction was observed between time and sex concerning handgrip strength in the right hand and standing long jump. Although positive effects of recreational team sports and self-exercise were noted within each sex independently, it became apparent that the magnitude of these differences could vary depending on sex. While definitively addressing this hypothesis poses challenges, potential explanations may be associated with neuromuscular factors, particularly in light of the observed interactions with muscular strength and power. Perhaps the presence of higher levels of muscular growth-promoting hormonal conditions or enhanced recruitment of neural drive may contribute to untrained men exhibiting advantages over untrained women, particularly in muscular development [65]. Nevertheless, there is a need for physiological-based research aimed at clarifying the mechanisms that underlie sex differences, offering insights into the variations in the magnitude of adaptations.

This study had some limitations that must be discussed. The first limitation refers to the fact that the participants were exposed to only 8 weeks of intervention. This circumstance could have limited the potential to observe greater improvements in aerobic, strength, and jump performance over time, as previously described [59]. Another limitation concerns the absence of monitoring the self-exercise control group during the exercise sessions, hindering a thorough comparison with the intensities experienced by the experimental groups. Moreover, it is important to note that without identifying the modifications in body composition, particularly concerning muscle mass and the adaptations of neural mechanisms, it is not possible to gain an insightful understanding of the factors that favoured the physical fitness adaptations in this study. Additionally, it is important to note the lack of inclusion of strength-oriented control groups as another limitation to consider. Although this could give a broader comparison to analyse to what extent the recreational team sports effects are similar to strength-oriented interventions, such methodology was outside the scope of this study. In future research, it is worth examining how recreational team games can elicit a higher cardiac response, enhance enjoyment, and elevate physical activity intensities, along with their impact on the rate of perceived exertion in comparison to other interventions [66].

## CONCLUSIONS

Recreational soccer and basketball activities have been found to significantly enhance overall cardiorespiratory health, handgrip strength (a general measure of muscle strength), and jump performance among sedentary, overweight, and obese young adults. Nevertheless, it is essential to take sex into account, especially concerning upper body strength and horizontal jump, when incorporating conditioned games. Although not significantly distinguished from the self-exercise control group, it was observed that group or individual physical exercise represents a significantly more effective approach for improving physical fitness compared to maintaining an inactive lifestyle. This opens avenues for exploring diverse options, especially for those who may not be particularly motivated towards individual exercise. Recreational team sports, in general, exhibit similarly favourable effects to individual sports, and innovative approaches that foster additional social engagement and motivation can improve long-term adherence. Community programmes and socially engaging activities, such as recreational team sports, could be promoted through public policies to mitigate the effects of sedentary lifestyles and enhance the quality of life for overweight and obese populations.
